# Molecular dynamics simulations of the glucocorticoid receptor DNA-binding domain suggest a role of the lever-arm mobility in transcriptional output

**DOI:** 10.1371/journal.pone.0189588

**Published:** 2017-12-15

**Authors:** Lautaro Damián Álvarez, Diego Martín Presman, Adalí Pecci

**Affiliations:** 1 Universidad de Buenos Aires, CONICET, UMYMFOR and Departamento de Química Orgánica, Facultad de Ciencias Exactas y Naturales, Buenos Aires, Argentina; 2 Laboratory of Receptor Biology and Gene Expression, Building 41, 41 Library Drive, National Cancer Institute, National Institutes of Health, Bethesda, MD, Unitec States of America; 3 Universidad de Buenos Aires, CONICET, IFIBYNE and Departamento de Química Biológica, Facultad de Ciencias Exactas y Naturales, Buenos Aires, Argentina; George Washington University, UNITED STATES

## Abstract

One of the first and essential steps in gene expression regulation involves the recruitment of transcription factors (TFs) to specific response elements located at enhancers and/or promoters of targeted genes. These DNA elements have a certain variability in both sequence and length, which may affect the final transcriptional output. The molecular mechanisms in which TFs integrate the subtle differences within specific recognition sequences to offer different transcriptional responses is still largely unknown. Here we used molecular dynamics simulations to study the DNA binding behavior of the glucocorticoid receptor (GR), a ligand-regulated TF with pleiotropic effects in almost all cells. By comparing the behavior of the wild type receptor and a well characterized Ala477Thr substitution within the rat GR DNA binding domain, we found that the region that connects the two-zinc fingers (i.e. the lever arm) would likely play a key role in GR transcriptional output.

## Introduction

The glucocorticoid receptor (GR) is a ligand-regulated transcription factor expressed in nearly all vertebrate cells. GR regulates, both positively and negatively, the expression of hundreds of genes in different cell types, involved in relevant processes such as metabolism, development, inflammation and the stress response, among others. The fact that GR can operate in a highly context-specific manner, yet being able to trigger defined specific cellular responses (e.g. the promotion of apoptosis in T-cells and the prevention of cell death in epithelial cells), raised the question of how this receptor in particular, and transcription factors in general, can achieve such precision and plasticity [1].

In the absence of ligand, the GR remains mainly cytoplasmic but upon hormone binding conformational changes in the receptor trigger its almost complete translocation into the nucleus, where it produces most of its biological functions. A direct GR mode of action involves the dynamic binding of the receptor to specific DNA sequences named Glucocorticoid Response Elements (GREs) [2]. Although the GR is widely believed to bind DNA as a homodimer, a recent study [3] suggests that the final active receptor would have a higher oligomeric structure *in vivo* [4].

**Fig 1:**
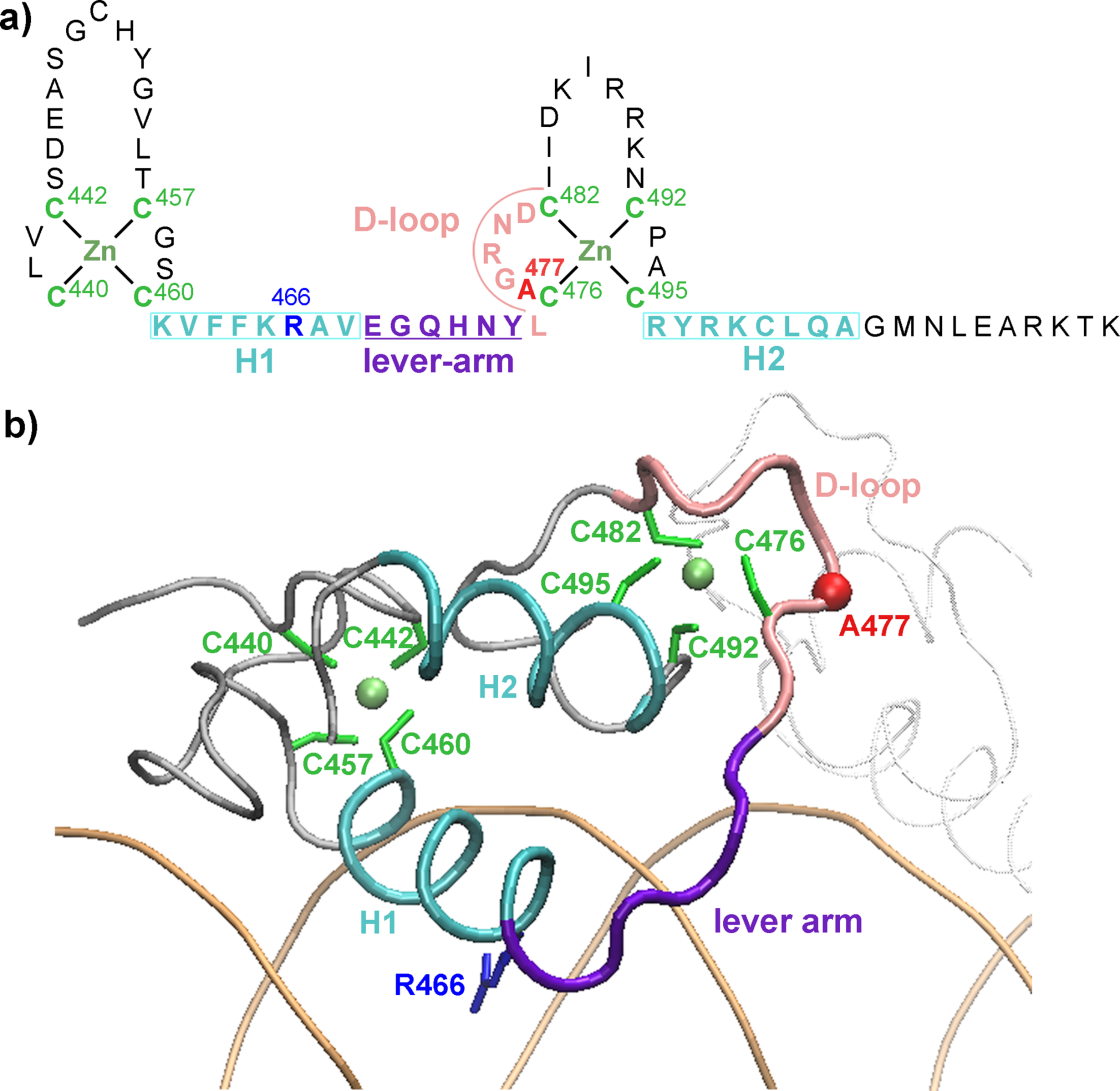
The GR DBD/Fkbp5 complex. a) Primary and secondary structure of the GR DBD. b) Representation of the crystal structure of the GR DBD/Fkbp5 complex (pdb:3g6p, chain A).

The GR is organized into three major domains: a poorly conserved N-terminal ligand-independent activation function-1 domain (AF-1), a highly conserved central DNA-binding domain (DBD) involved in specific DNA motifs recognition, and a C-terminal ligand-binding domain (LBD), responsible for ligand binding and interaction with several co-factors [5]. GR’s DBD consists of two zinc finger modules that differs each other in their conformation and function (Fig 1) [6]. Each module has four Cys residues coordinating a single Zn^++^, followed by an amphipathic helix and a peptide loop. The H1 helix of the first zinc-finger module, also referred as the “DNA-reading helix” [1], contains the Arg466 residue that makes base-specific contacts in the major groove of the DNA binding sequence. In the second module, the H2 helix makes nonspecific contacts with the DNA backbone at the minor groove, whereas the peptide loop (D-loop) provides monomer–monomer contacts, stabilizing the formation of GR DBD dimers on DNA. A “lever arm” region loop connects both zinc-finger modules [7]. Point-mutations at the D-loop have been shown to affect GR’s transcriptional activity [8–10].

The best-characterized GR–DNA interaction was described on the canonical GRE, which is composed of two pseudo-palindromic hexameric AGAACA repeats, separated by a three-base-pair spacer [11]. Based on crystal structures and NMR studies [7, 12], it has been proposed that the spacer sequences could alter DNA shape, resulting in conformational changes that allosterically propagate throughout the lever arm and alter the conformation of the receptor’s D-loop, ultimately resulting in the GR transcriptional activity modulation.

Among the many relevant functions of glucocorticoids in general homeostasis, the most pharmacological exploited action is their powerful anti-inflammatory activity. Interestingly, a single point-mutation at the D-loop, Ala to Thr at position 458 in human, 465 in mouse and 477 in rat (Fig 1) -the GRdim mutant- has played a major role during more than two decades of research in the search for safer glucocorticoid drugs (reviewed in [13]). Although the GRdim was originally thought to be devoid of dimerization and DNA binding, and consequently being incapable to directly modulate transcriptional activity [9, 10]; later, accumulated evidence demonstrated that this mutant forms dimers [14–16] and binds to DNA in vitro [17] and *in vivo* [14, 15, 18, 19]. However, GRdim’s transcriptional output differs greatly from its wild type counterpart and, in many cases, it is mostly unable to directly modulate gene expression [7, 14, 20, 21]. Therefore, at present, the molecular mechanisms behind the different phenotypic behavior between both GRwt and GRdim still remain to be elucidated.

Here, by using molecular dynamics simulations (MD), we compared the behavior of both GR wt and GRdim (Ala477Thr) DBD’s when recruited to DNA. Our main aim is to understand at the molecular level the consequences of introducing a hydroxyl and additional methyl group within the DBD’s dimerization region. We chose to run the simulation on two different GREs, Fkbp5 and Pal-F enhancers, that differ each other only at their spacer region but, interestingly, they provoke opposite transcriptional responses. When these sequences drive the expression of a minimal promoter in U2OS cells, Fkbp5 induced expression is higher when the GRwt is recruited, while Pal-F induces more expression with the GRdim mutant [12] (Table 1). Results from 100 ns of MD simulations provide precise information on the dynamic behavior of both complexes, mainly at the lever-arm and D-loop regions that could explain the differences between GRwt and GRdim transcriptional activities.

**Table 1:** Simulated GR DBD/DNA complexes.

System	Receptor	Protein Chains	DNA sequence	Fold induction^a^
**S1**	GRwt	A, B	Fkbp5: AGAACAgggTGTTCT	4.6
**S1A**	GRwt	A	Fkbp5: AGAACAgggTGTTCT	nc
**S1B**	GRwt	B	Fkbp5: AGAACAgggTGTTCT	nc
**S2**	GRdim (Ala477Thr)	A, B	Fkbp5: AGAACAgggTGTTCT	2.7
**S2A**	GRdim (Ala477Thr)	A	Fkbp5: AGAACAgggTGTTCT	nc
**S2B**	GRdim (Ala477Thr)	B	Fkbp5: AGAACAgggTGTTCT	nc
**S3**	GRwt	A, B	Pal-F: AGAACAaaaTGTTCT	3.0
**S4**	GRdim (Ala477Thr)	A, B	Pal-F: AGAACAaaaTGTTCT	4.6

^a^Data extracted from Ref. [12]

## Results

### Monomeric versus dimeric GR DBD/DNA complexes

**Fig 2:**
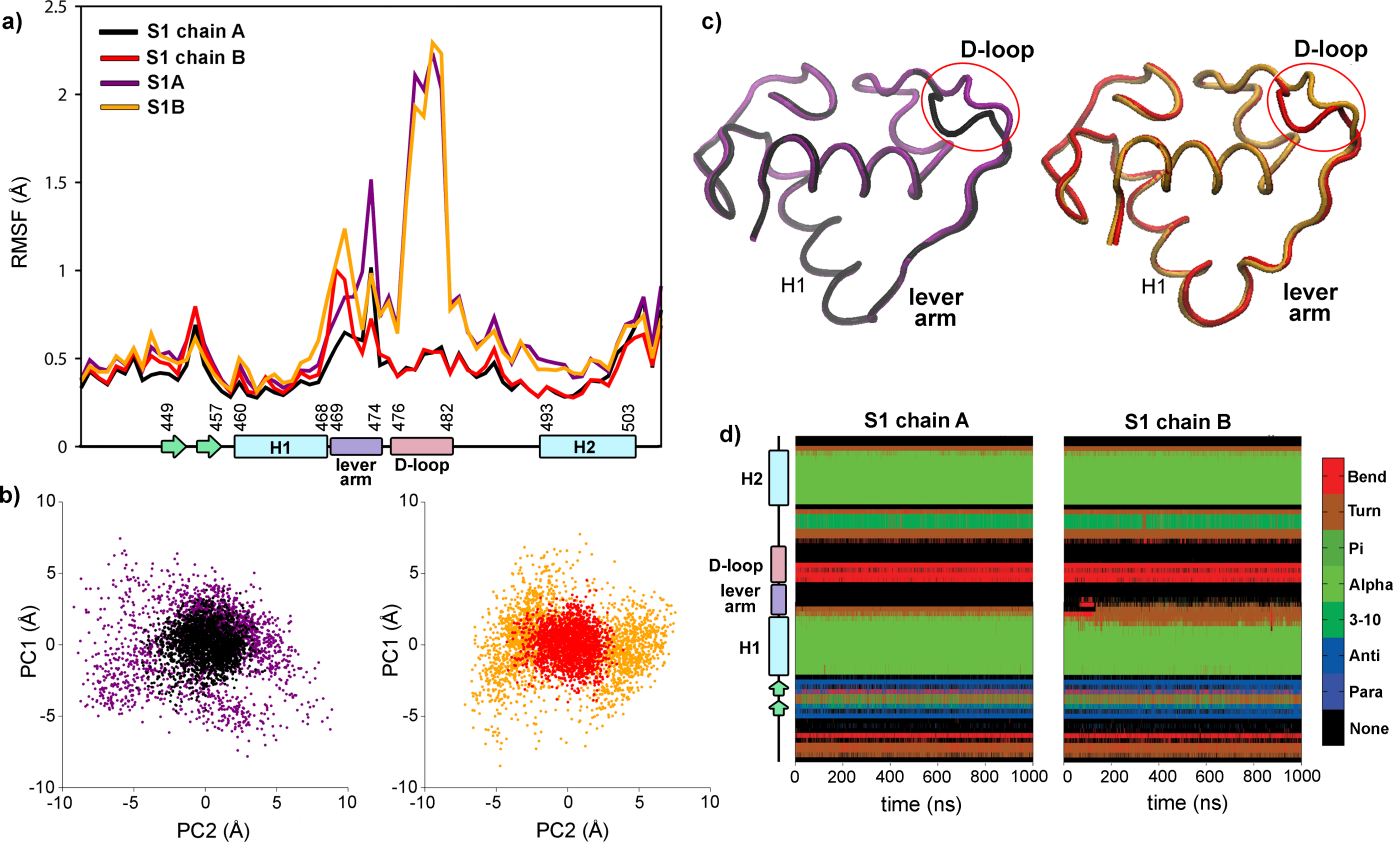
The absence of the GR DBD dimer partner provokes a strong dynamical alteration on the D-loop. a) RMSF values of the S1 system, chain A in black and chain B in red, S1A system in purple and S1B system in yellow. The secondary structure of the GR DBD is schematized along the x-axis. b) Principal component analysis of the MD trajectories: projection on the first two eigenvectors in S1, chain A in black and chain B in red, S1A system in purple and S1B system in yellow. c) Average structures of S1, chain A in black and chain B in red, S1A system in purple and S1B system in yellow. d) Time evolution of secondary structure propensities for the S1 system.

As a first approximation to investigate the dynamic behavior of the D-loop residues, we compared both monomeric and dimeric GRwt DBD/DNA complexes. S1A and S1B systems (Table 1) were constructed by simple deletion of chain B or chain A atoms of S1 structure, respectively, and then all complexes were simulated by 100 ns MD. Visual inspection of the trajectories shows that the protein and DNA global folding remains essentially intact in all cases, with RMSD values between 1.0 and 2.0 Å (S1 Fig). The RMSD plots of the monomeric systems (S1A and S1B) showed more fluctuations than the dimeric one. Moreover, the Root Mean Square Fluctuation (RMSF) of alpha carbons, which provide a time-average representation of per-residue fluctuations, displays marked differences among the systems (Fig 2A). Clearly, a much larger fluctuation of the D-loop, an in a lesser extent of the lever-arm, is observed in both monomeric systems compared to the dimeric one. In the S1A and S1B systems, the D-loop is the region with most mobility, being fluctuations higher than 2 Å, while in the S1 system the D-loop remains stabilized. When the principal component analysis (PCA) was performed over the backbone atoms of these trajectories, a marked difference was detected among the configurational space sampled for these complexes (Fig 2B). The monomeric S1A and S1B systems explore a more extended area than the dimeric S1 system, indicating that the absence of the dimer partner provokes a strong dynamical alteration on the GR DBD structure. Indeed, a comparison of the time-averaged structures of each system indicates that the conformation of the D-loop is strongly stabilized in both chains upon dimerization (Fig 2C).

On the other hand, regarding the behavior of the lever arms in the S1 system, a small yet different RMSF pattern was observed, with the N-terminal end of chain A or the C-terminal end of chain B reaching values close to 1 Å (Fig 2A). Moreover, when the temporal evolution of the secondary structure propensity was analyzed using the DSSP method, a loss of helicity in the C-terminal residues of the H1 was observed in the chain B. Thus, the H1 resulted in two residues shorter than the chain A (Fig 2D), having these residues a larger mobility. It is noteworthy that structural asymmetries between the dimer partners were also inferred by previous analysis of several GR DBD/DNA crystal structures [7] and by NMR experiments [12], indicating that the asymmetry plays a functional role in GR action.

To understand the effects of the Ala477Thr mutation on the GR DBD structure, we constructed S2, S2A and S2B mutant systems from the S1 system (Table 1) by simply replacing the alanine for threonine side chain atoms. Again, monomeric systems showed more fluctuations of the RMSD values (S1 Fig) and a marked increment of the D-loop mobility was also observed (S2 Fig), while no major differences were found among the behavior between the mutant and wt monomeric complexes (S2 Fig). Therefore, these findings suggest that the structural changes that alters the transcriptional activity should be occurring when the GRdim dimerizes on the Fkbp5 sequence.

### GRwt versus GRdim dimeric GR DBD/DNA complexes

#### The role of the residue 477 on the dimerization interface

**Fig 3:**
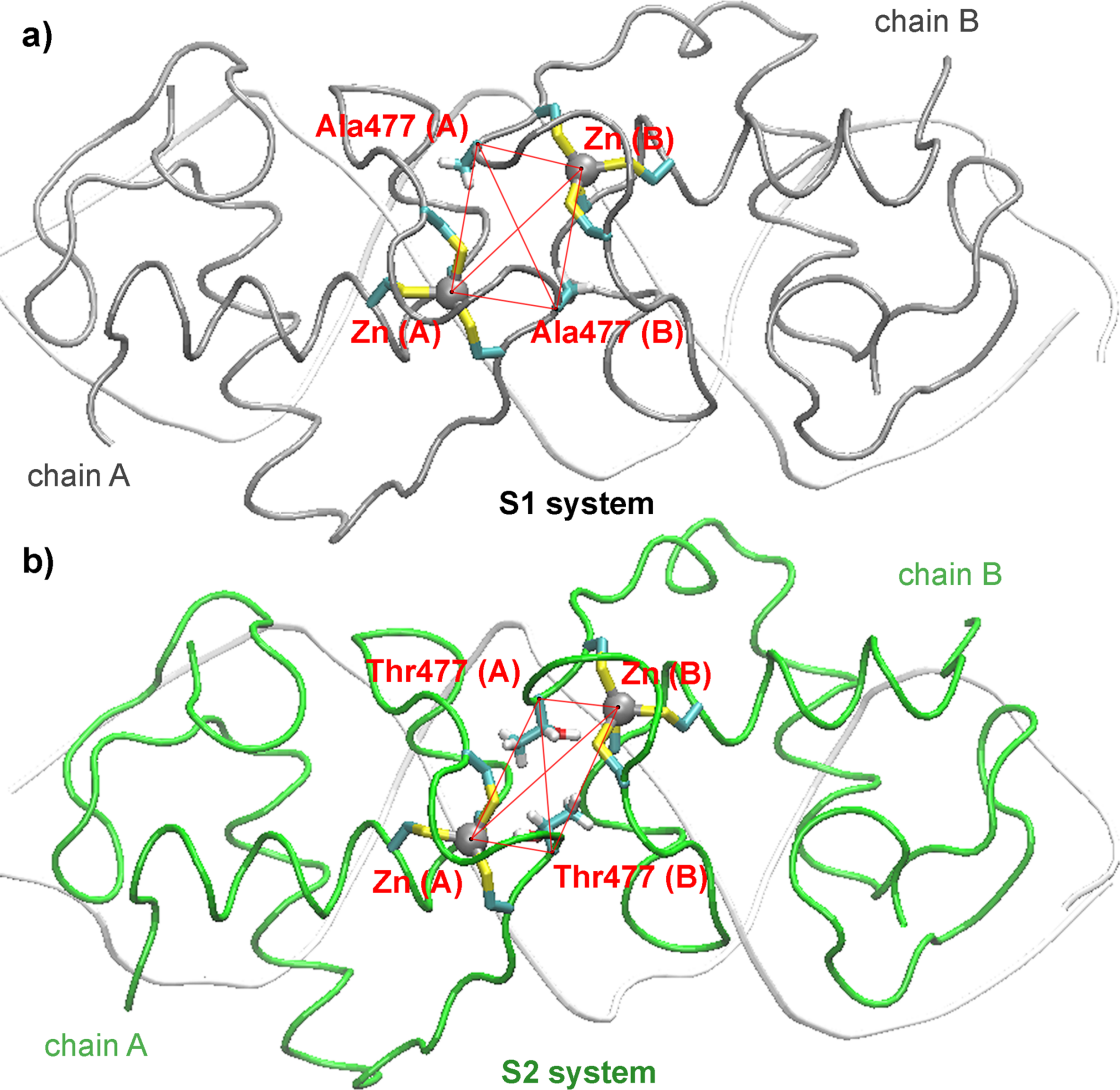
The A477T mutation affects the dimerization interface. Overall view of the average structures of the S1 (a) and S2 (b) systems showing the location of the dimerization zinc fingers and residues 477.

An overall view of the time-averaged structures of the dimeric wild type S1 and mutant S2 complexes is showed in Fig 3A and 3B, respectively, where the localization of the D-loops, the associated zinc fingers and the residues 477 are highlighted. The comparison of these structures reveals key alterations in the dimerization interface. As a measurement of these changes, average distances among the zinc atoms of the dimerization zinc finger motifs and CA atoms of residues 477 were calculated (Table 2). Clearly, a shortening of distances between the CA of residues 477 and zinc atoms of the same monomer is observed in the mutant system, while an increase of the distances involving CA and Zn atoms from different monomers occurred. Besides, while the distance between zinc atoms is larger in the mutant systems, the distance between CA atoms of residues 477 resulted smaller. In fact, an abrupt shortening of distance after 4 ns between methyl groups of the side chains of residues 477 is observed, which suggests that a hydrophobic contact between these residues was reached (S3 Fig). By monitoring the torsion angle formed by CA of residue 477 (A), Zn (A), CA of residue 477 (B) and Zn (B) atoms (Fig 3), we found that in the S1 systems these atoms remain almost coplanar (5°). Instead, in the mutant system a pronounced deviation was observed, with a torsion angle fluctuating around 28°.

**Table 2:** The A477T mutation alters the GR DBD dimerization interface.

	Systems			
	S1	S2	S3	S4
**Distance (Å)**^**a**^				
Zn (chain A)–CA Thr477 (chain A)	5.8 ± 0.3	4.6 ± 0.4	5.7 ± 0.4	4.6 ± 0.3
Zn (chain B)–CA Thr477 (chain B)	5.8 ± 0.2	4.5 ± 0.2	5.8 ± 0.2	4.5 ± 0.1
Zn (chain B)–CA Thr477 (chain A)	7.7 ± 0.3	8.3 ± 0.4	7.8 ± 0.3	8.3 ± 0.3
Zn (chain A)–CA Thr477 (chain B)	7.7 ± 0.2	8.3 ± 0.4	7.8 ± 0.3	8.2 ± .3
Zn (chain A)–Zn (chain B)	9.5 ± 0.2	10.5 ± 0.2	9.8 ± 0.1	10.6 ± 0.2
CA Thr477 (chain A)–CA Thr477 (chain B)	9.8 ± 0.3	7.9 ± 0.4	9.4 ± 0.4	7.8 ± 0.3
**Hydrogen Bond Occupancy (%)**^**b**^				
Asp481 (chain A)—Arg479 (chain B)	90	70	92	77
Asp481 (chain B)—Arg479 (chain A)	91	73	91	68
Ile483 (chain A)—Ala/Thr477 (chain B)	68	47	70	53
Ile483 (chain A)—Ala/Thr477 (chain B)	66	50	61	50
Leu475 (chain A)—Asn491 (chain B)	86	60	76	68
Leu475 (chain B)—Asn491 (chain A)	81	54	68	52
Thr477 (chain A)—Asn491 (chain B)	nc	92	nc	91
Thr477 (chain B)—Asn491 (chain A)	nc	82	nc	89
**MM (Kcal/mol)**^**c**^				
vdw	-47.5 ± 3.1	-45.3 ± 3.6	-49.9 ± 4.2	-49.8 ± 4.6
ele	-50.8 ± 13.8	-34.0 ± 15.2	-55.8 ± 16.2	-35.6 ± 14.8
Total	-98.3 ± 13.4	-79.3 ± 15.0	-105.7 ±16.1	-85.4 ± 14.5

^a^Average distances among zinc atoms of the dimerization Zn finger and the CA atoms of residues 477.

^b^Hydrogen Bond Occupancy of inter-monomeric interactions.

^c^Inter-monomer interaction energy contributions to the total energy of the MM force field computed using the MM/PBSA method (vdw: Van der Waals; ele: electrostatic; Total: total MM binding energy). PBSA terms were ignored.

**Fig 4:**
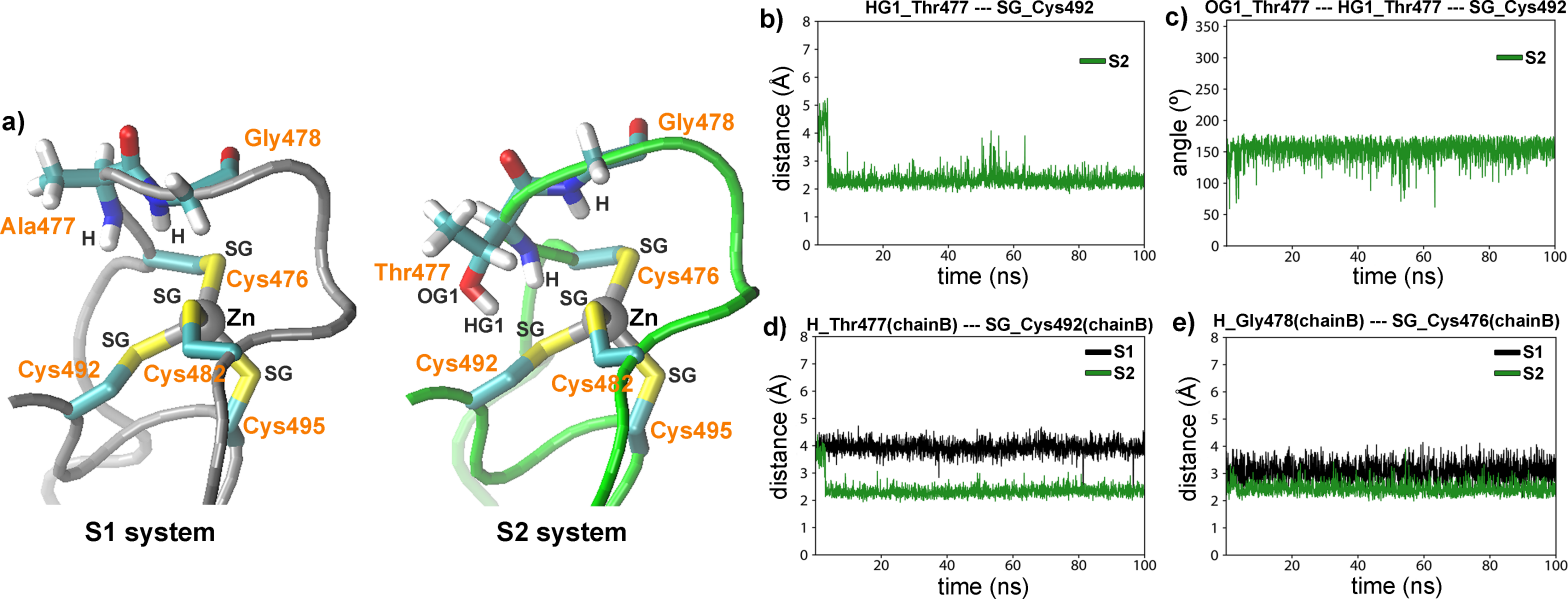
Thr477 forms hydrogen bond interactions with residues of the dimerization zinc finger that alters its structure. a) Detailed view of the average structures of the dimerization zinc fingers (chain A) in S1 and S2 systems. b) Time evolution of the distance between the HG1 atom of residue Thr477 (chain A) and the SG atom of Cys492 in the S2 system (chain A). c) Time evolution of the angle between OG1 and HG1 atoms of Thr477 and SG atom of Cys492 in the S2 system (chain A). d) Time evolution of the distance between the H atom of residue 477 and SG atom of Cys492 in S1 and S2 systems (chains A). f) Time evolution of the distance between H atom of Gly478 and SG atom of Cys476 in S1 and S2 systems (chains A).

On the other hand, hydrogen bond interactions between the hydroxyl of Thr477 and its polar neighbor residues were also calculated. Results from chain A show that, after an initial readjustment of the conformation of the side chains, a very stable configuration is achieved, in which the hydroxyl moiety of Thr477 is re-oriented to the sulfur atom of the Cys492 (Fig 4A). The temporal evolution of the distance between the HG hydrogen atom of Thr477 and the SG sulfur atom of Cys492 reveals that after 4 ns, the distance is stabilized with an average value of 2.2 Å (Fig 4B). Concomitantly, the OG1 (Thr477)-HG1(Thr477)-SG(Cys492) angle exhibits a similar evolution (Fig 4C), with an averaged value of 160°. In this way, both distance and angle values suggest the formation of a hydrogen bond among the HG1(Thr477) and the SG(Cys492). A very similar behavior was observed in the chain B of the S2 system (S4 Fig). Remarkably, a recent analysis on crystalline structures of proteins containing cysteine or methionine residues showed that hydrogen bonds involving acceptor sulfur atoms have an averaged geometry very similar to those found here [22].

In addition to the above intra-monomer contact, the hydroxyl groups of Thr477 take part of an inter-monomer interaction with the side chain of the Asn491 partner (S5 Fig). Distances obtained between these atoms indicate the formation of a strong and stable hydrogen bond, with an average value of 2.1 Å.

According to NMR studies carried out by Watson et al [12], the largest difference in amide backbone signals in the wild type vs mutant GR DBD/Fkbp5 complexes was detected in the Cys492, Ala/Thr477 and Gly478 residues. Consistently, the MD results show that this region of the D-loop is exactly where a large conformational change is observed when the S1 and S2 systems are compared (Fig 4A). The time evolution of the distance between the H hydrogen atom of Ala/Thr477 and the SG sulfur atom of Cys482 (Fig 4D), and between the H hydrogen atom of Gly478 and the SG sulfur atom of Cys476 (Fig 4F) showed that in the mutant complex (S2) the distances are extensively smaller than in the wild type (S1) complex. This modification in the chemical environment of these residues is consistent with the NMR data interpretation. Similar results were obtained when MD results of the chains B of the S1 and S2 systems were compared (S6 Fig). Furthermore, in order to study the reproducibility of the MD simulations, we obtained an independent run (50 ns) of the S2 system starting from a different initial condition. The analysis of the new S2 simulation shows similar results than that previously obtained. Remarkably, all hydrogen bonds involved in the Thr477 of the S2 system are also formed in the new simulation performed with the mutant receptor. As an example, the time evolution of the distance between the HG1 atom of residue Thr477 and the SG atom of Cys492 are shown in the S7 Fig.

Thus, we concluded that both hydroxyl and methyl group of Thr477 participate directly in durable and strong interactions that influence the conformation of the mutant lever-arm and D-loop.

#### Dimerization interface in GR DBD/DNA complexes

**Fig 5:**
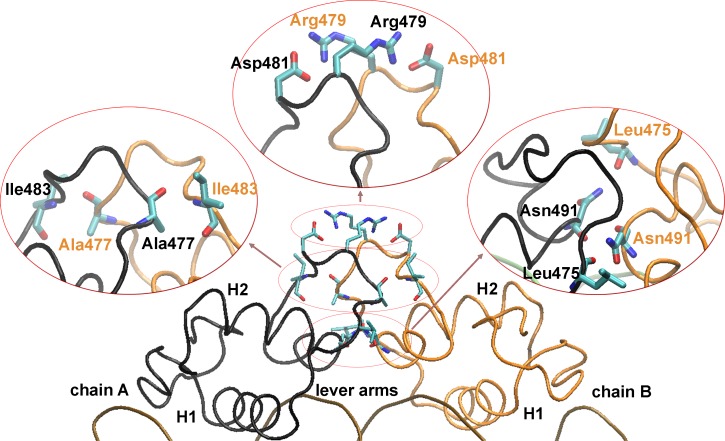
The A477T mutation affects polar inter-monomeric interactions. Average structure of the S1 system showing the main polar interaction between chain A and chain B residues.

Once characterized the local modifications produced by the Ala477Thr mutation on the D-loop, we next analyzed the dimerization interface of the GR DBD by determining the polar interactions between both monomers. The analysis of hydrogen bonds occupancy (HBO) reveals three major groups of inter-monomeric interactions in the S1 system (Fig 5): a pair of hydrogen bonds between the backbone atoms of Leu475 and the side chain of Asn491, a pair of salt bridges between the side chain of Arg479 and Asp481, and a pair of hydrogen bonds between the backbone atoms of Ala/Thr477 and Ile483. Table 2 shows that the HBO of these three groups exceed 50% in both S1 and S2 systems. However, overall the wild type values are considerably larger than the mutant ones, indicating a more persistent interaction between wild type monomers compared to GRdim monomers. In fact, when the MM/PBSA method was used to compute the energetic contributions from the electrostatic energy and Van der Waals interactions, the sum of which gave the total MM binding energy, we found that the electrostatic contribution is *ca*. 15 Kcal/mol smaller in the wild type than in the mutant system (Table 2), while the Van der Waals contributions resulted similar. In this way, despite the additional inter-monomeric interaction formed between Thr477 and the Asn491 partner, the total polar contacts were reduced in the mutant complex.

#### Protein-DNA interactions and geometric analysis of DNA

Once described the conformational changes produced in the lever-arm and the D-loop by the Ala477Thr mutation, we wonder whether DNA spacer sequence would influence the conformational structure. We chose the Fkbp5/Pal-F pair since they generate opposite transcriptional responses upon activation of the GRwt and GRdim receptors [12]. Therefore, the S3 and S4 systems were constructed from the S1 and S2 systems, respectively, by replacing the three central guanines by adenines to obtain the corresponding dimeric GR DBD/Pal-F complexes (Table 1). MD results show stable trajectories for S3 and S4 systems (S1 Fig), in which the global DNA conformation is maintained during the whole simulation scale. Regarding the D-loop and the dimerization interface, the Pal-F complexes preserved the global conformation found in the corresponding Fkbp5 systems, with similar average distances, calculated HBO and electrostatic and Van der Waals contributions (Table 2). Besides, all hydrogen bonds involving the dimerization zinc finger atoms and Thr477 are conserved in S4, with similar average distance values (S3, S4, S5 and S6 Figs).

To evaluate the putative differences in the mode in which GR DBD molecules recognize the Fkbp5 and the Pal-F sequences, we compared the HBO protein-DNA interactions among the four systems. Results showed similar values for all systems, with the major polar interaction formed between arginines of H1 and H2 helices and the guanines at level ±3 (S8 Fig and S1 Table). The Arg466 (H1) forms two specific contacts with the nitrogenated base, one of them almost always present (with the O6 atom) and other less frequent (with the N7 atom). Arg496 and 498 maintain nonspecific interactions with the phosphate backbone groups.

**Fig 6:**
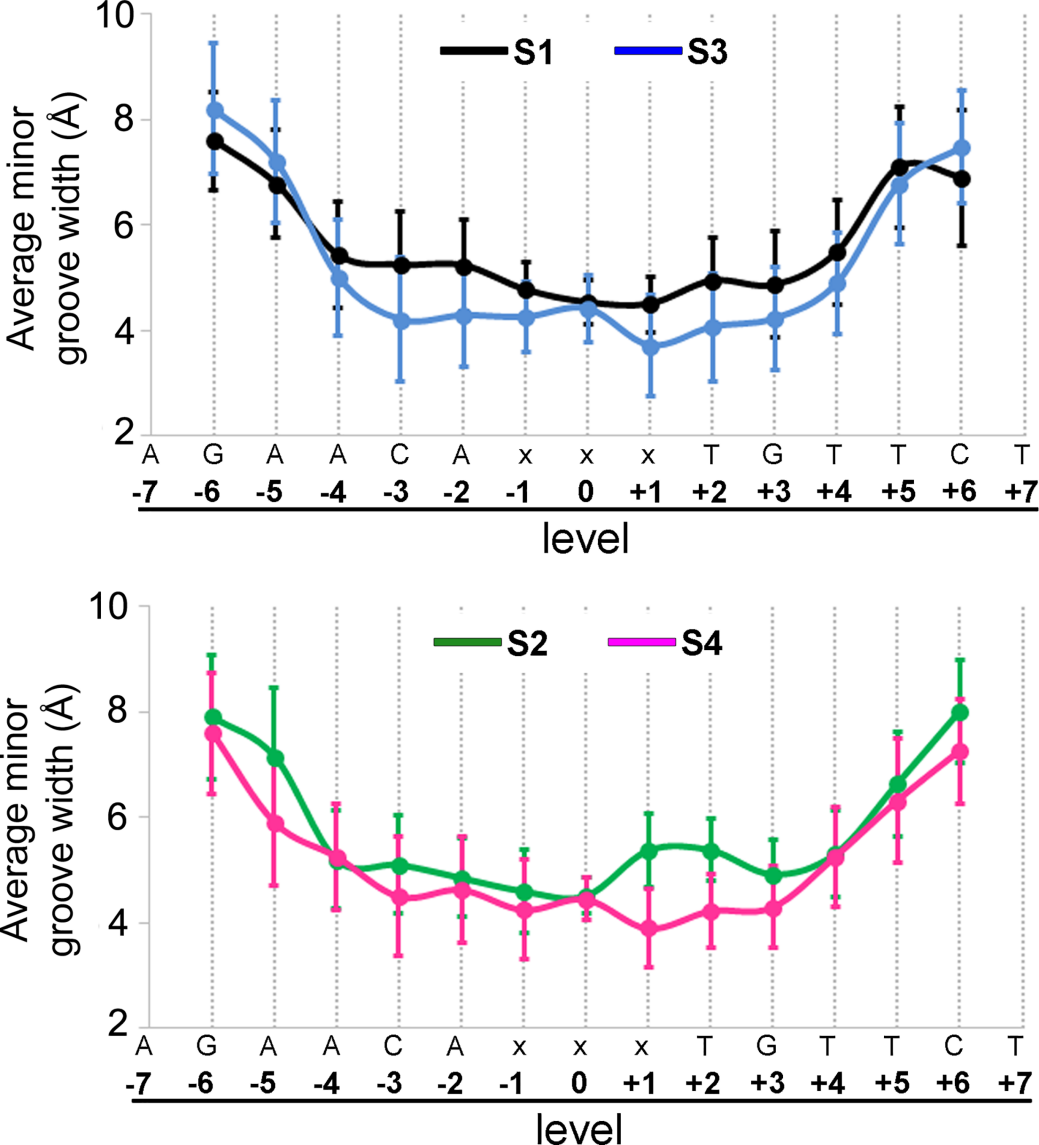
The GRE spacer affects the minor groove width. Variation of the average minor groove width along the oligomer in S1 (black), S2 (green), S3 (blue) and S4 (magenta) (x = G for S1 and S3 systems, x = A for S2 and S4 systems).

To investigate changes in the DNA conformation produced by the different spacer sequences of Fkbp5 and Pal-F systems, we determined the geometric DNA parameters. While no major differences among the systems were found for the global curvature and the average major groove width, the average minor groove width clearly varies among systems (Fig 6). Results showed that, even though in the center of the spacer (level 0) all systems have similar values, the adjacent pairs (levels -2, -1, +1 y +2) have important differences. Globally, the Pal-F systems (AAA spacer) show a narrower minor groove than Fkbp5 systems (GGG spacer). The temporal evolution of the minor groove width for these levels showed that Fkbp5 molecules have larger values than the Pal-F ones, especially where the DNA contacts the chain B residues of the GR DBD (levels +1 and +2) (S9 Fig). These MD results are consistent with those derived from crystal structures, and from previous studies showing that short A-tracts narrow the minor groove [12, 23].

**Fig 7:**
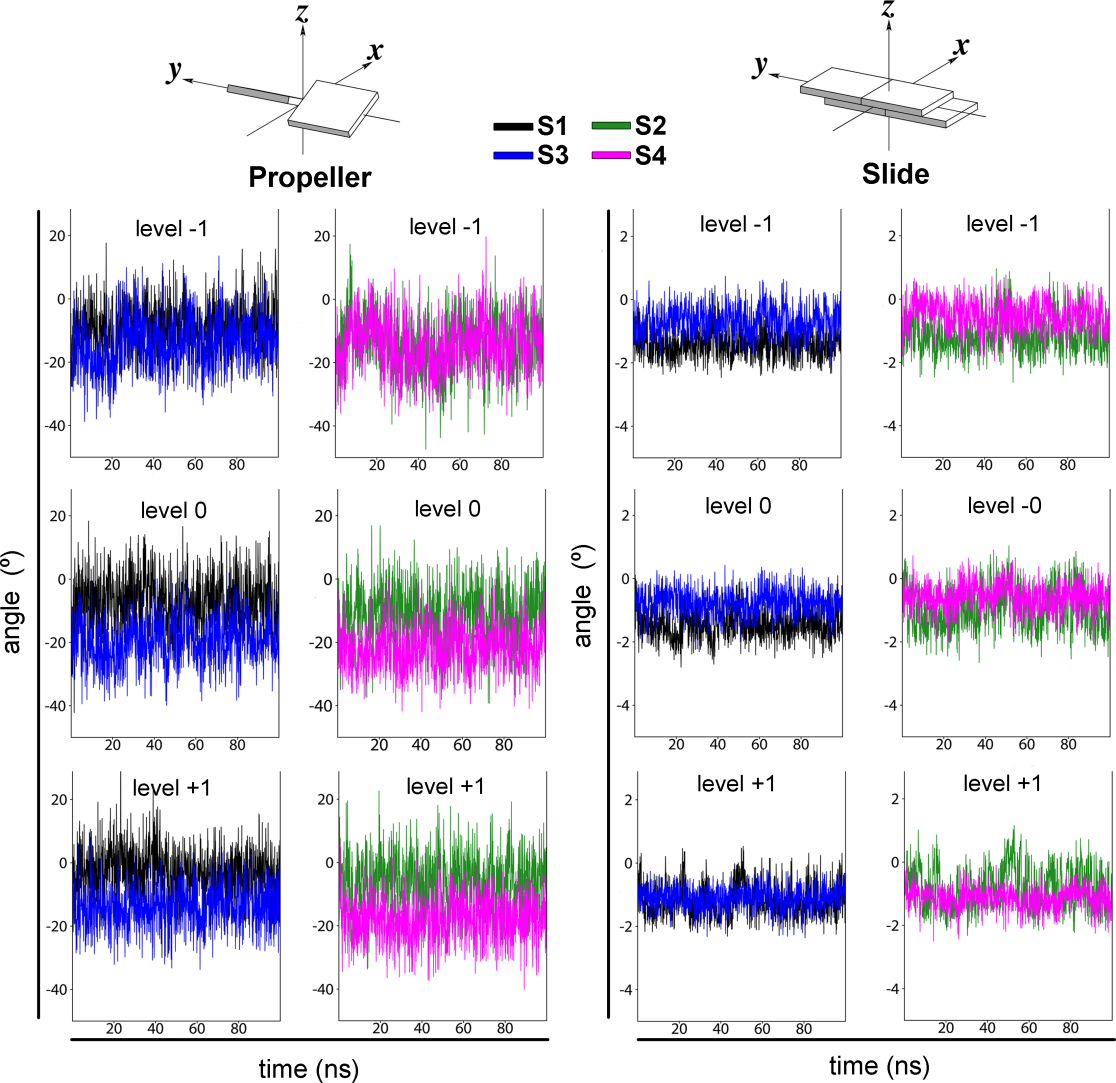
The GRE spacer sequence alters the propeller and the slide base-pair parameters. Time evolution of propeller and slide values for S1 (black), S2 (green), S3 (blue) and S4 (magenta) systems. Schematic representations were extracted from Ref. [24].

Finally, base pair parameters were calculated for all dimeric complexes, finding substantial differences among Fkbp5 and Pal-F systems mainly in the “slider” and “propeller” values of levels -1, 0 and 1 (Fig 7). Thus, we conclude that the spacer sequence, not only affects the minor groove width, but also alters the relative disposition of the DNA bases.

#### Dynamical behavior of dimeric GR DBD/DNA complexes

**Fig 8:**
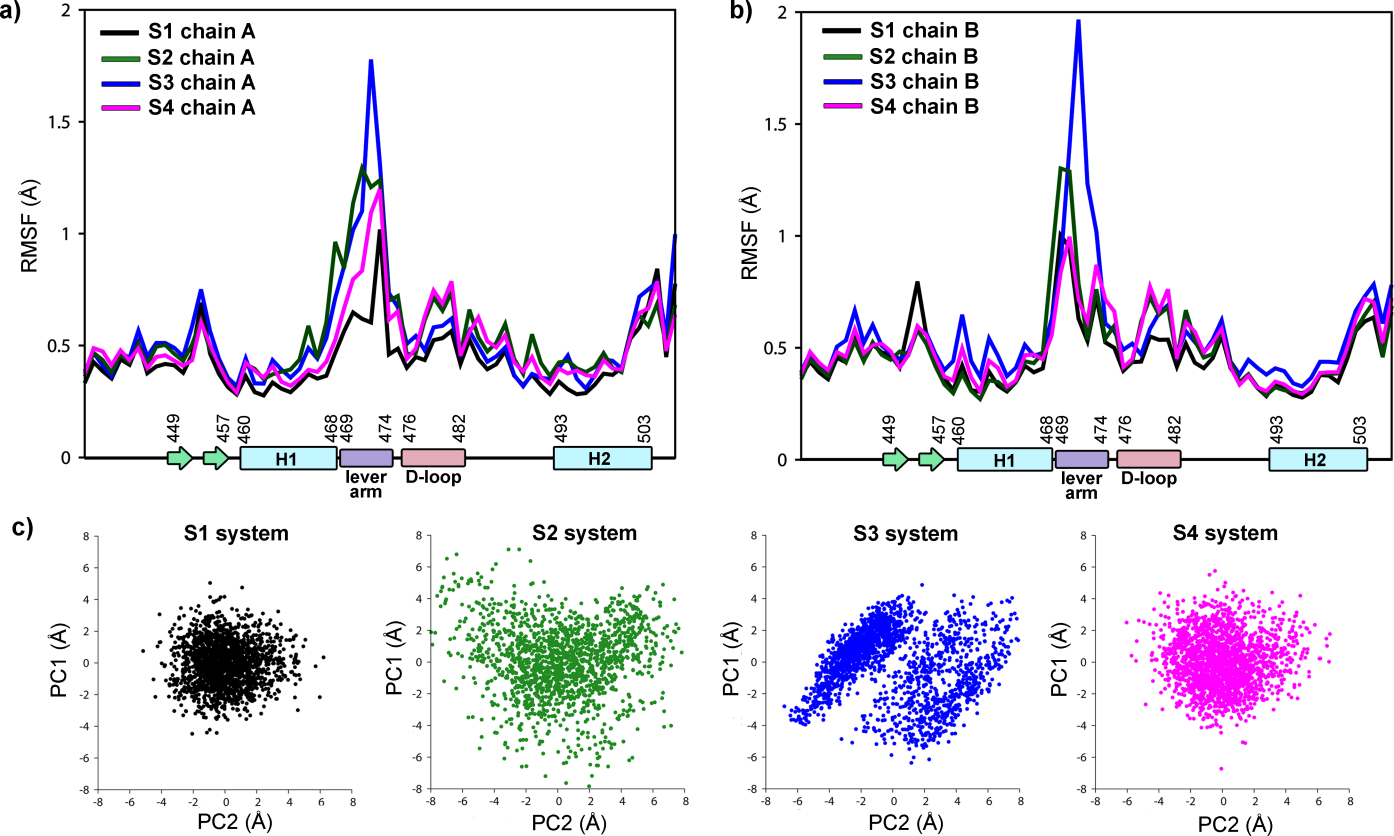
Complexes that are more active have lever-arms with a reduced mobility. a) RMSF values of chain A residues of S1, S2, S3 and S4 systems. b) RMSF values of chain B residues of S1, S2, S3 and S4 systems. The secondary structure of GR DBD is schematized along the x-axis. c) Principal Component Analysis of MD trajectories: projection on the first two eigenvectors of lever arm and D-loop CA atoms in S1, S2, S3 and S4 systems.

As an initial step to compare the dynamic behavior of the S1, S2, S3 and S4 systems, we calculated the RMSF of alpha carbon atoms of the GR DBD (Fig 8A and 8B). In all complexes, the lever-arm is clearly the more fluctuating region. It reached the maximal mobility in the S3, followed by the S2, and lastly by the S1 and S4 systems. Consistently, the PCA calculation applied over the lever-arm and D-loop alpha carbon atoms reveals that a larger configurational space is explored in the S2 and S3 systems (Fig 8C).

**Fig 9:**
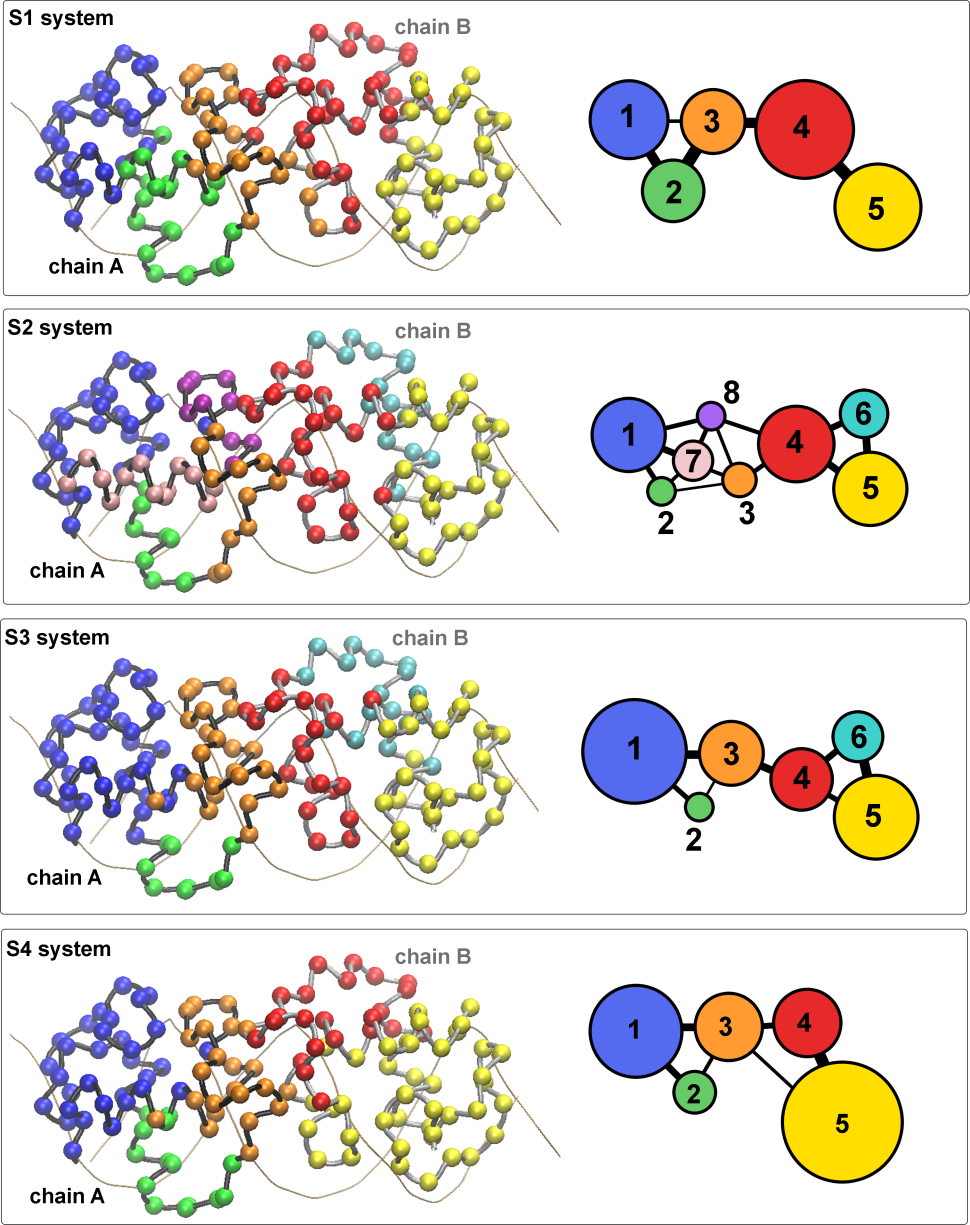
The less active complexes have an altered community structure. Community structure of the GR DBD (chains A and B) displayed in the three-dimensional structure (a) or in schematic two-dimensional representation (b). The two-dimensional view of the communities depicts the relative size of the communities (number of residues) as colored circles of varying areas and the relative interconnectivity strength as lines of varying thicknesses.

To gain more insight into the allosteric mechanisms involved in the different transcriptional activities of the GR DBD/DNA complexes, we have used a community network analysis to identify groups of residues (communities) that are closely correlated. The resulting communities (c_i_) of alpha carbon atoms (nodes) are depicted in Fig 9. First, we observed variations in the total number of communities. For the S1 and S4 systems, 5 communities were found, while more communities are formed in the S2 and S3 complexes (8 and 6 communities, respectively). Second, a different distribution of nodes is obtained on each system. In the S1, S3 and S4 complexes, the nodes of chain A belong to one of three communities (c_1_, c_2_ or c_3_), with the lever-arm completely in c_2_ and D-loop entirely in c_3_. In contrast, two additional communities (c_7_ and c_8_) are observed for the chain A nodes of the S2 system. These findings indicate a less correlated movement of chain A residues in the S2 system. Nevertheless, even more substantial differences are found for the chain B nodes. Two communities (c_4_ and c_5_) exist in the S1 and S4 complexes, while the other systems (S2 and S3) have an additional community (c_6_). Thus, a clear difference exists between the more active complexes (S1 and S4) and the less active ones (S2 and S3). Interestingly, the more active systems have the lever-arm and D-loop taking part of the same community (c_4_), while in the less active complexes these regions belong to separate communities (the lever-arm in c_6_ and the D-loop in c_4_).

## Discussion

As the original characterization of the GRdim mutant (inability to form dimers and bind to DNA [9, 10]) provided a simple explanation of its phenotype, later work has proven a much complex scenario [4, 7, 12, 14, 15, 21]. Now the question remains: How the single exchange between Ala and Thr within the DBD alters the transcriptional response of GR? In this work, we carried out a set of MD simulations of GR DBD/DNA complexes to gain insight into the allosteric mechanism involved in the GRwt and GRdim actions. We believe that our findings may offer an explication at molecular level of the main conclusions derived from NMR experiments [12], about how the A477T mutation affects the surrounding residues and the dimerization loop, and also how protein and DNA changes are integrated to modulate GR’s transcriptional activity.

We initially showed that the presence of the dimer partner drastically modified the dynamic behavior of the D-loop, demonstrating that dimerization of GR’s DBD proceed with a marked conformational change on this fundamental region, which in turn, results in strong polar interactions between the monomers. The analysis of the GRdim DBD/DNA complexes revealed that the hydroxyl group of the Thr477 side chain takes part of two stable hydrogen bonds, one intra-monomeric (with the Cys491) and the other inter-monomeric (with the Asn471). These perdurable interactions were accompanied with marked changes in the conformational state of the D-loop, mainly in residues surrounding the dimerized zinc finger. Notably, these findings are consistent with previous NMR results showing that this region within GR’s DBD is the most affected by the Thr477Ala mutation [12]. In fact, NMR experiments clearly showed that there are only three residues (Ala/Thr477, Gly478 and Cys492, all belonging to the dimerization zinc finger) that present relevant differences in the amide backbone signals when the wt and mutant GR DBDs were compared. This undoubtedly indicates that the A477T mutation produces a local receptor reorganization that modifies the chemical environment around the backbone atoms of these residues. In this sense, our modeling results offer for first time a precise picture of how these three residues could be reorganized in the GR DBD mutant. The changes produced by this mutation significantly diminished the hydrogen bond formation and the electrostatic contribution to the total MM energy of the inter-monomer interaction. Since the transcriptional activity of GR complexes also depend on the DNA sequence, the deficient DBD-DBD contacts observed in the mutant systems cannot be directly associated to a lesser activity. However, the reduced inter-monomeric interaction in the S2 system could explain the smaller cooperativity observed in the electrophoretic mobility shift assays [12], where a strong positive cooperativity was observed for the wild type DBD while the GRdim DBD nearly saturated the DNA as a monomer before dimer formation. It still remains to be tested whether this positive cooperative effect is biologically relevant *in vivo* when the entire GR receptor and not only the DBD fragment is evaluated.

The examination of the dynamic behavior of dimeric DBD complexes does contribute to understand the relative transcriptional activity of the studied systems. NMR results have shown that the A477T mutation generates local structural changes as well as structural reorganization in regions outside of the dimerization surface [12]. Chemical-shift differences in the N-terminal region of the lever arm and in the recognition helix of wild type and mutant GR DBD were observed, supporting the idea that the dimerization and DNA interfaces are structurally coupled [12]. MD simulations showed that the more active complexes have lever-arms with a reduced mobility compared to the less active ones. Moreover, the chain B of the most active complexes exhibits a coupled movement of lever-arm and the D-loop residues, while in the less active complexes a disruption between these fundamental regions is observed. We also show that the replacement of the GGG by the AAA spacer does not alter the polar interactions between the protein and the DNA, consistently with the fact that transcriptional induction of GR does not vary as a simple function of DNA affinity [12]. However, the minor groove width and some base pair parameters of the spacer and adjacent zones are considerably affected.

In summary, we have found that a) the identity of the D-loop residue 477 (Ala or Thr) establishes how GR DBD molecules dimerize and b) the identity of the spacer (GGG or AAA) determines the DNA shape. Collectively, from the coupling of these dimerization and DNA “states”, complexes with variable transcriptional activity could arise. The lever-arm appears to be the region involved in the allosteric mechanism that integrates the signals coming from the DNA/DBD to other protein domains, as previously speculated [7]. In this sense, the way the lever arm “responds” to the DNA sequence in the wild type or the mutant receptor may alter co-factor interactions at the LBD or AF-1 level, ultimately leaving to different transcriptional outcomes. Since there is no structural evidence to date supporting information about how the DBD connects the other GR domains or co-factors, it is not possible to know how subtle local changes at the DNA/DBD level reverberate into the receptor *in toto* and affect the final GR response. Our recent report, focused on mapping the GR dynamics within the nucleus of living cells, concluded that the GRdim/Dex spatial distribution, its affinity to the NCoA-2 co-factor, and its dynamics at binding sites resemble those observed for the wild-type GR/Dex complex [25]. However, two main differences between this mutant and the wild type receptor have been detected: its lifetime at chromatin longer-lived sites and its NCoA-2-dependent redistribution. Particularly, the GRdim mutation would affect the residence time of the coactivator at GR chromatin targets. This impaired interaction may be related to the inability of this mutant to upregulate gene expression. Despite further work is required to determine whether the inability of GRdim to be allosterically modulated by DNA influences GRdim-NCoA-2 interaction, we believe that our MD simulations contribute understand the allosteric mechanism that integrates the signals coming from the DNA and the dimerization interface.

## Materials and methods

### Starting GR DBD/DNA structures

Taking into account the previous experimental studies with the rGR DBD/DNA complexes [7, 12], the initial coordinates for the MD simulations were taken from the X-ray crystal structure pdb:3g6p, resolution: 1.99 Å (Fig 1B). The GRwt DBD/DNA complex (S1) consists of an 18 bp palindromic DNA fragment of the Fkbp5 enhancer and a GR DBD dimer (Table 1). The chain B (Ser437-Arg510) has seven residues more than chain A (Ser437-Lys517), which are folded as α-helix (H3). These residues and all the crystallographic water molecules were conserved. The PROPKA method was used to calculate the protonation states of ionizable residues [26] and then hydrogen atoms were added using the Tleap program [27]. The system was immersed in an octahedral box of TIP3 water molecules [28] with the solute molecules at least 15 Å from the box boundaries. Then 56 Na^+^ and 16 Cl^-^ counterions were added using the Tleap program in order to satisfy the electroneutrality and the experimental ionic strength (100 mM). The S1A and S1B were directly obtained by deletion of chain B or chain A atoms of S1 structure, respectively. The S2, S2A and S2B complexes were obtained from the S1 by removing the corresponding Ala477 side chain and introducing the threonine side chain (GRdim) with the Tleap program. The S3 and S4 initial coordinates were obtained from the equilibrated S1 and S2 structures, respectively, by replacing the GGG spacer with the AAA spacer using the 3DNA program [24].

### Molecular dynamics

Molecular dynamics were performed with the AMBER 14 software package [28]. The FF14SB force field parameters were used for GR DBD [29]. and the parm99+bsc1 was used for DNA [30]. The force field parameters and RESP charges of the zinc centers were referenced from the previous work [31]. All systems were initially minimized for 10000 steps with a harmonic restraint (50 kcal/mol/Å^2^) on protein and DNA atoms, and then 10000 steps of unrestrained minimization were carried out. The energy-minimized systems were heated throughout two sequential steps of 500 picoseconds. First, systems were heated to 200 K at constant volume. Then temperature was slowly increased at constant pressure to the desired production temperature (300 K). In both steps a restraint (10 kcal/mol/Å^2^) fixing the backbone protein and DNA atoms was applied. Finally, 250 picoseconds were carried out at 1 atm and 300 K in which the restraint on the protein backbone and DNA atoms was gradually reduced to zero. Starting from these equilibrated structures, 100 ns MD production runs were performed. The production runs were done at 300 K in a NPT ensemble using periodic boundary conditions and the particle mesh Ewald method (grid spacing of 1 Å) for treating long-range electrostatic interactions with a uniform neutralizing plasma. Temperature regulation was performed using a by Langevin thermostat with a collision frequency of 2 ps^−1^. The pressure was controlled with the Berendsen barostat using a pressure relaxation time of 1 ps. The SHAKE algorithm was used to keep bonds involving H atoms at their equilibrium length, allowing the use of a 2 fs time step for the integration of Newton’s equations with the velocity Verlet algorithm.

### Analysis of the results

MD trajectories were analyzed with the CPPTRAJ module [32] and representative snapshots were obtained using VMD [33]. Only residues 440 to 506 of the GR DBD were considered for the analysis. The root mean squared deviation (RMSD), the time evolution of the distances among selected atoms, the time evolution of torsion angles between selected atoms and the time evolution of the secondary structure (calculated by using the DSSP method [34]) were monitored over the complete production trajectory (10000 snapshots). The first 10 ns of trajectories were discarded for the root mean square fluctuations (RMSFs), the Principal Component Analysis (PCA), the Hydrogen Bond Occupancy (HBO), the MM-PBSA calculation and the Dynamical Network Analysis. Therefore, only the last 9000 snapshots were used. Hydrogen bond occupancy was calculated using the default parameters for the distance cutoff (acceptor to donor heavy atom less than 3.0 Å) and for the angle cutoff (135°). The MM/PBSA.py tool [35] implemented in AMBER was used to compute the electrostatic and van der Waals contributions to the total energy of the molecular mechanics (MM) force field. The PCA [36] was performed by diagonalizing a variance-covariance matrix of the system 3 N atomic positional fluctuations in order to obtain a new set of coordinates (eigenvectors) to describe the system motions. Each eigenvector (or principal component, PC) has an associated eigenvalue corresponding to the mean square fluctuation contained in the system’s trajectory projected along that eigenvector. By sorting the eigenvectors according to their eigenvalues, the first principal components (PC1 and PC2) correspond to the system’s highest amplitude motion. The Dynamical Network Analysis [37, 38], a method of characterizing allosteric signaling through biomolecular complexes, was applied to MD simulations using the NetworkView plugin of the VMD. Networks were constructed by defining all CA protein atoms in a system as nodes. Any pair of nodes that reside within a 4.5 Å cutoff for more than 75% of the MD trajectory is connected via an edge, with the weight of the edge being proportional to the covariance between the nodes. Then, networks were resolved into communities, which are groups of nodes with correlated motions, using the Girvan-Newman algorithm [39]. The magnitude of communication flow between communities was quantified by the total betweenness of all edges that transition between communities. Geometric DNA analysis and base pair parameters were obtained using Curves+ [40] and Canal utility.

## Supporting information

S1 Table Hydrogen Bond Occupancy between GR DBD and DNA atoms. (DOCX)

S1 Fig Stability of the MD simulations. Root mean squared deviation (RMSD) from the initial structures measured over the backbone atoms of all simulated systems.
(TIF)

S2 Fig The absence of the GR DBD dimer partner provokes a strong dynamical alteration on the D-loop. a) RMSF values of the S2 system, chain A in black and chain B in red, S2A system in purple and S2B system in yellow. b) RMSF values of the S1A in black and S2A in green. c) RMSF values of the S1A in black and S2B in green. The secondary structure of the GR DBD is schematized along the x-axis. d) Time evolution of secondary structure propensities for the S1 system.
(TIF)

S3 Fig Hydrophobic contact between methyl sidechain atoms of Thr477. Time evolution of distances between CG2 carbons of Thr477 in S2 and S4 systems.
(TIF)

S4 Fig Hydrogen bond interaction between Thr477 and Cy492. a) Time evolution of the distance (a) or the angle (b) between indicated atoms of S1 and S4 systems.
(TIF)

S5 Fig Intramonomer hydrogen bond interaction between Thr477 and Asn491. Detailed view of the average structures of the S2 system showing the intramonomer contacts between these residues. Time evolution of distances between oxygen OG1 atom of Thr477 and hydrogen HD22 atom of Asn491 in S2 and S4 systems.
(TIF)

S6 Fig Hydrogen bond interaction between Thr477 and residues of the dimerization zinc finger. Time evolution of distances between indicated atoms of S1 and S4 systems. See Fig 3D.
(TIF)

S7 Fig Hydrogen bond interaction between Thr477 and Cy492. Time evolution of the distance between indicated atoms of the replica of the S2 system.
(TIF)

S8 Fig Hydrogen bond interactions between the GR DBD and the DNA. Representative structure of the S1 system showing the major polar interactions among GR DBD and DNA atoms.
(TIF)

S9 Fig Minor groove widths of DNA. Time evolution of the minor groove width in level -2 to +2 for S1 (black), S3 (blue), S2 (green) and S4 (magenta) systems.
(TIF)
