# Supplementary material for: Molecular dynamics simulations of the glucocorticoid receptor DNA-binding domain suggest a role of the lever-arm mobility in transcriptional output
Source: PLoS One. 2017 Dec 15;12(12):e0189588. doi: 10.1371/journal.pone.0189588 (PMC5731742; doi:10.1371/journal.pone.0189588)
Supplement: S1 Table — (DOCX) [file pone.0189588.s001.docx]

|  | **System** | | | |
| --- | --- | --- | --- | --- |
|  | **S1** | **S2** | **S3** | **S4** |
| **Hydrogen Bond Occupancy (%)** |  |  |  |  |
| Arg466/NH2 (chain A) - G/O6 (level -3) | 94 | 87 | 93 | 95 |
| Arg466/NH2 (chain B) - G/O6 (level +3) | 90 | 94 | 94 | 91 |
| Arg466/NH1 (chain A) - G/N7 (level -3) | 38 | 44 | 51 | 47 |
| Arg466/NH1 (chain B) - G/N7 (level +3) | 42 | 40 | 43 | 46 |
| Arg496/NH1 (chain A) - G/OP2 (level -3) | 96 | 84 | 89 | 81 |
| Arg496/NH1 (chain B) - G/OP2 (level +3) | 98 | 98 | 96 | 93 |
| Arg489/NE (chain A) - G/OP1 (level -3) | 91 | 83 | 96 | 87 |
| Arg489/NE (chain A) - G/OP1 (level -3) | 96 | 96 | 96 | 94 |
