# Supplementary figures and images for: Molecular dynamics simulations of the glucocorticoid receptor DNA-binding domain suggest a role of the lever-arm mobility in transcriptional output
Source: PLoS One. 2017 Dec 15;12(12):e0189588. doi: 10.1371/journal.pone.0189588 (PMC5731742; doi:10.1371/journal.pone.0189588)

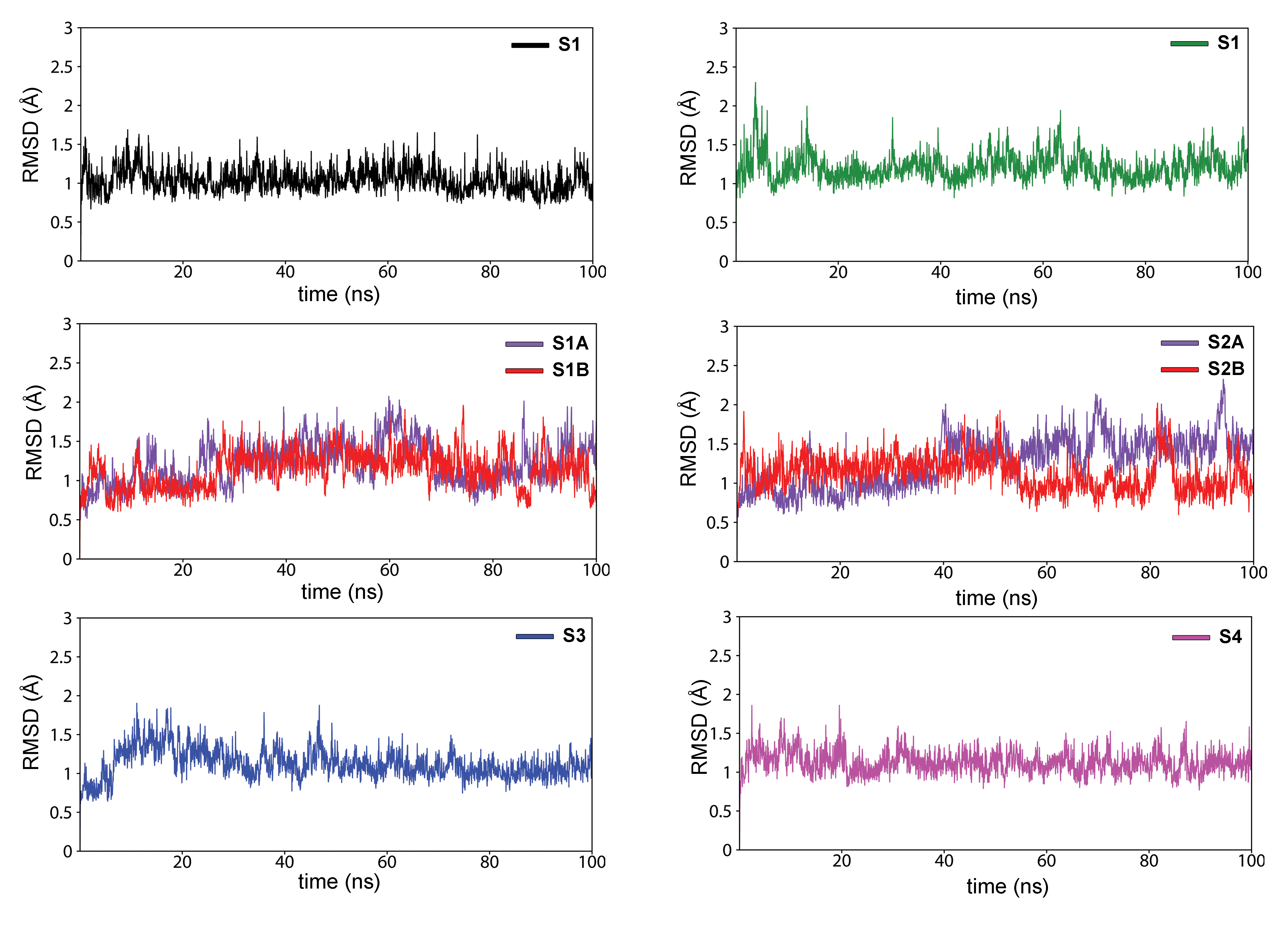

Supplement: S1 Fig — Root mean squared deviation (RMSD) from the initial structures measured over the backbone atoms of all simulated systems. (TIF) [file pone.0189588.s002.tif]

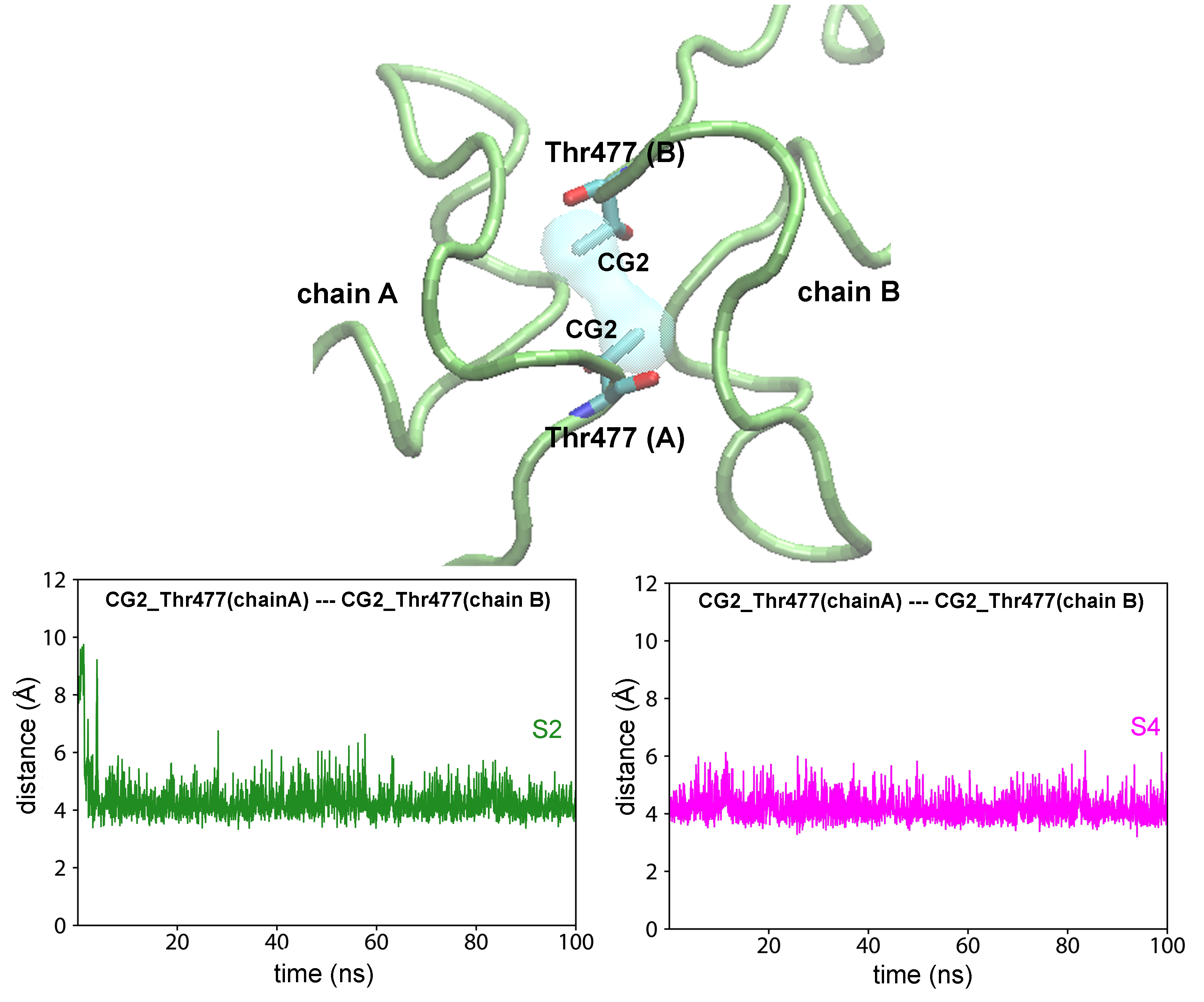

Supplement: S3 Fig — Time evolution of distances between CG2 carbons of Thr477 in S2 and S4 systems. (TIF) [file pone.0189588.s004.tif]

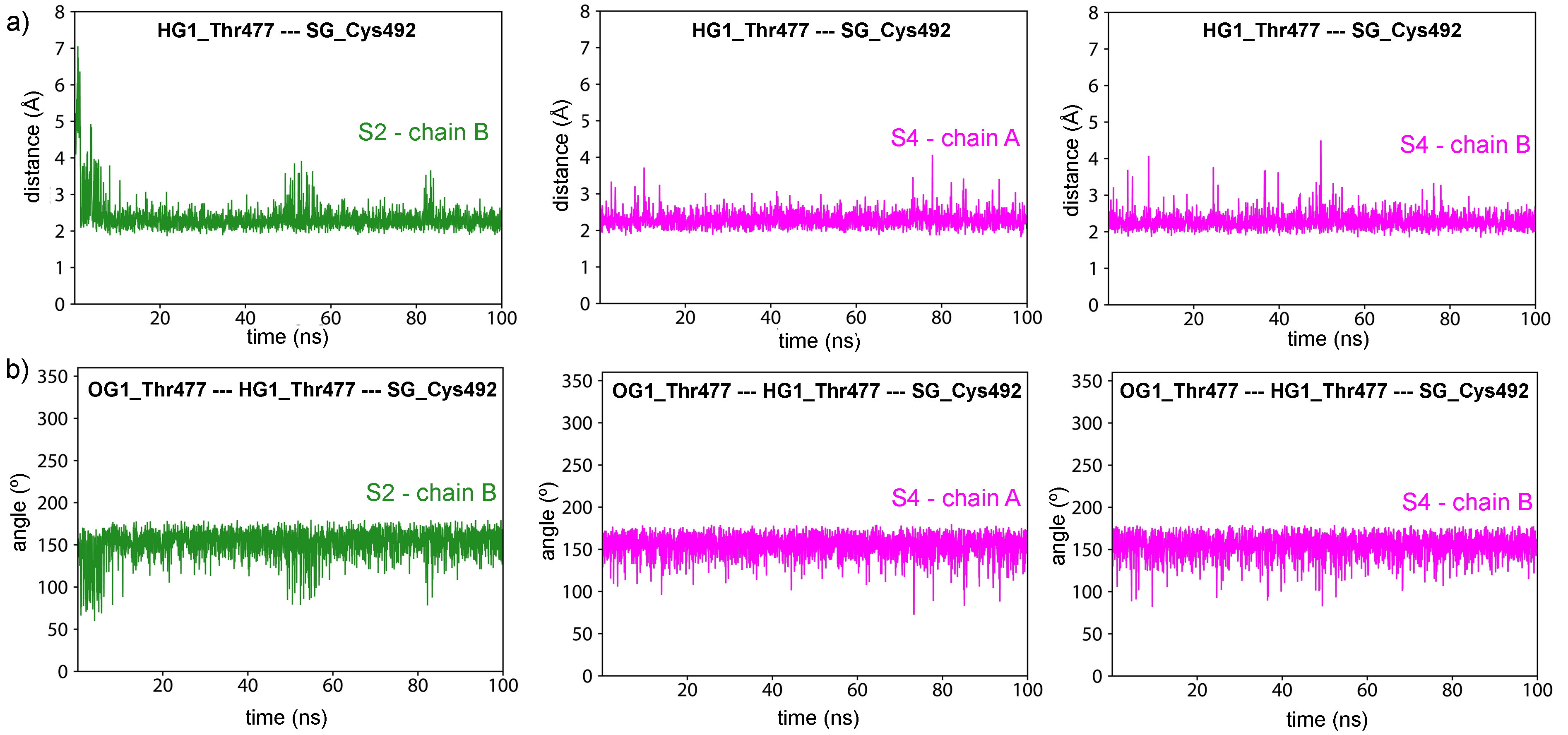

Supplement: S4 Fig — a) Time evolution of the distance (a) or the angle (b) between indicated atoms of S1 and S4 systems. (TIF) [file pone.0189588.s005.tif]

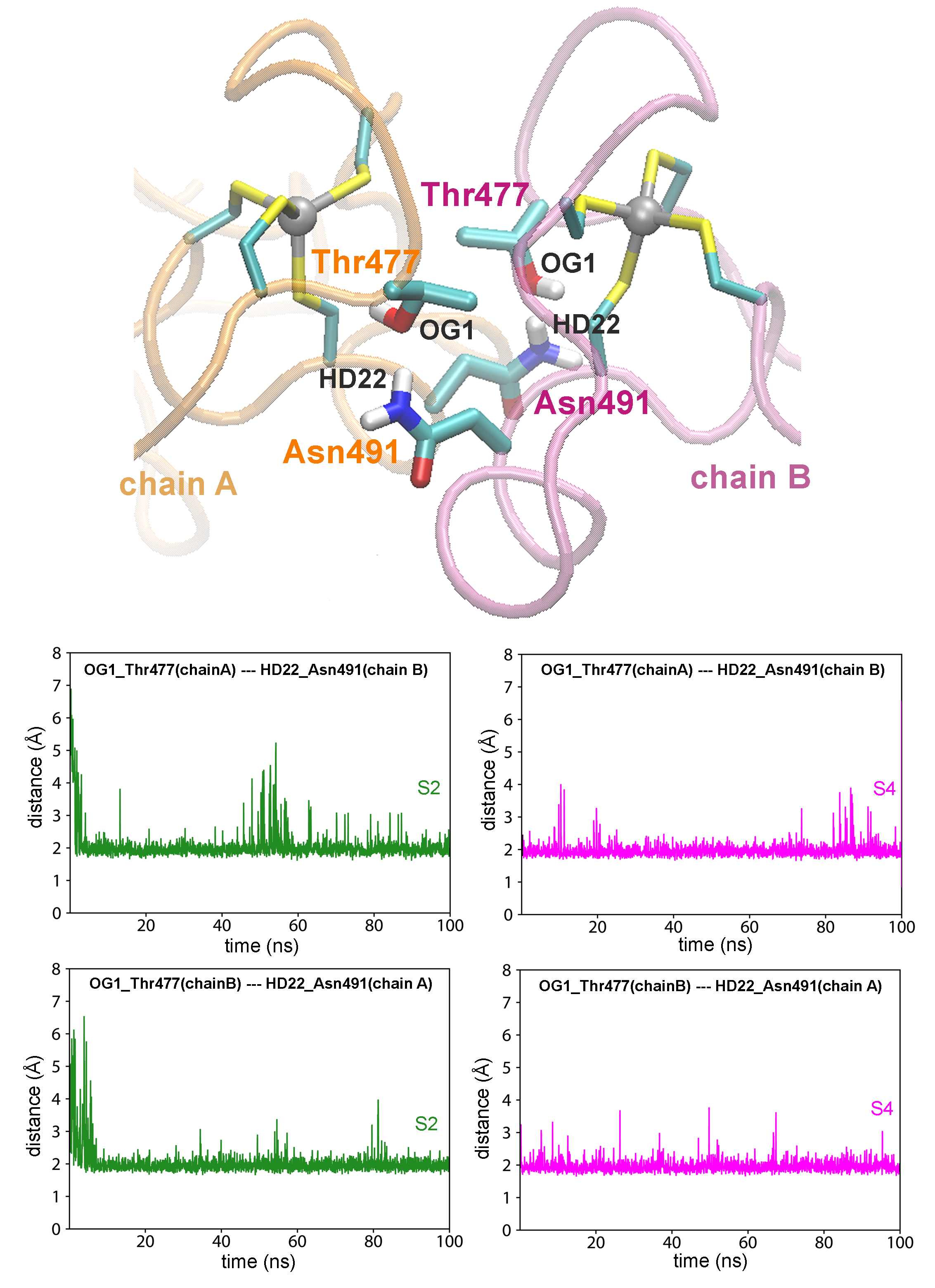

Supplement: S5 Fig — Detailed view of the average structures of the S2 system showing the intramonomer contacts between these residues. Time evolution of distances between oxygen OG1 atom of Thr477 and hydrogen HD22 atom of Asn491 in S2 and S4 systems. (TIF) [file pone.0189588.s006.tif]

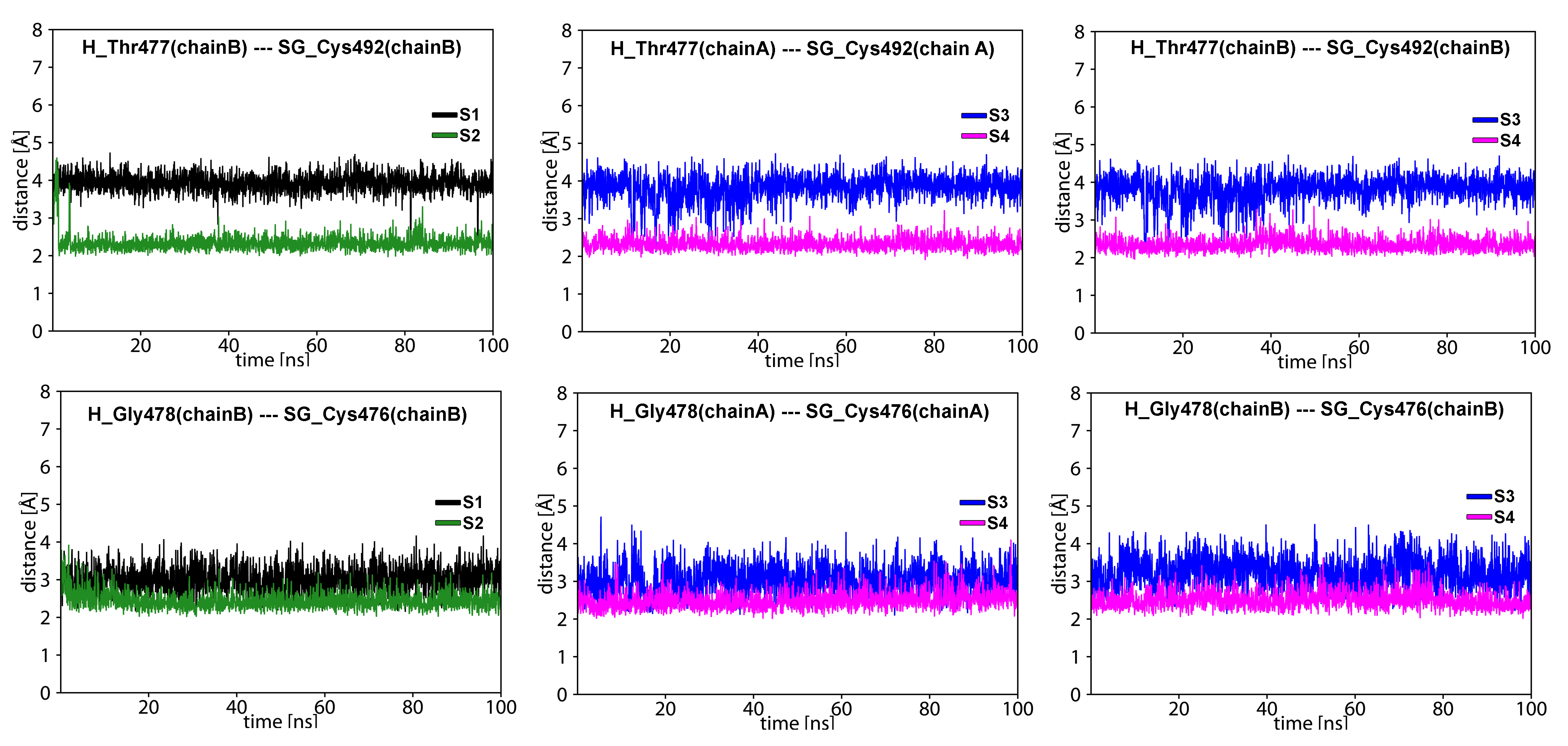

Supplement: S6 Fig — Time evolution of distances between indicated atoms of S1 and S4 systems. See Fig 3D. (TIF) [file pone.0189588.s007.tif]

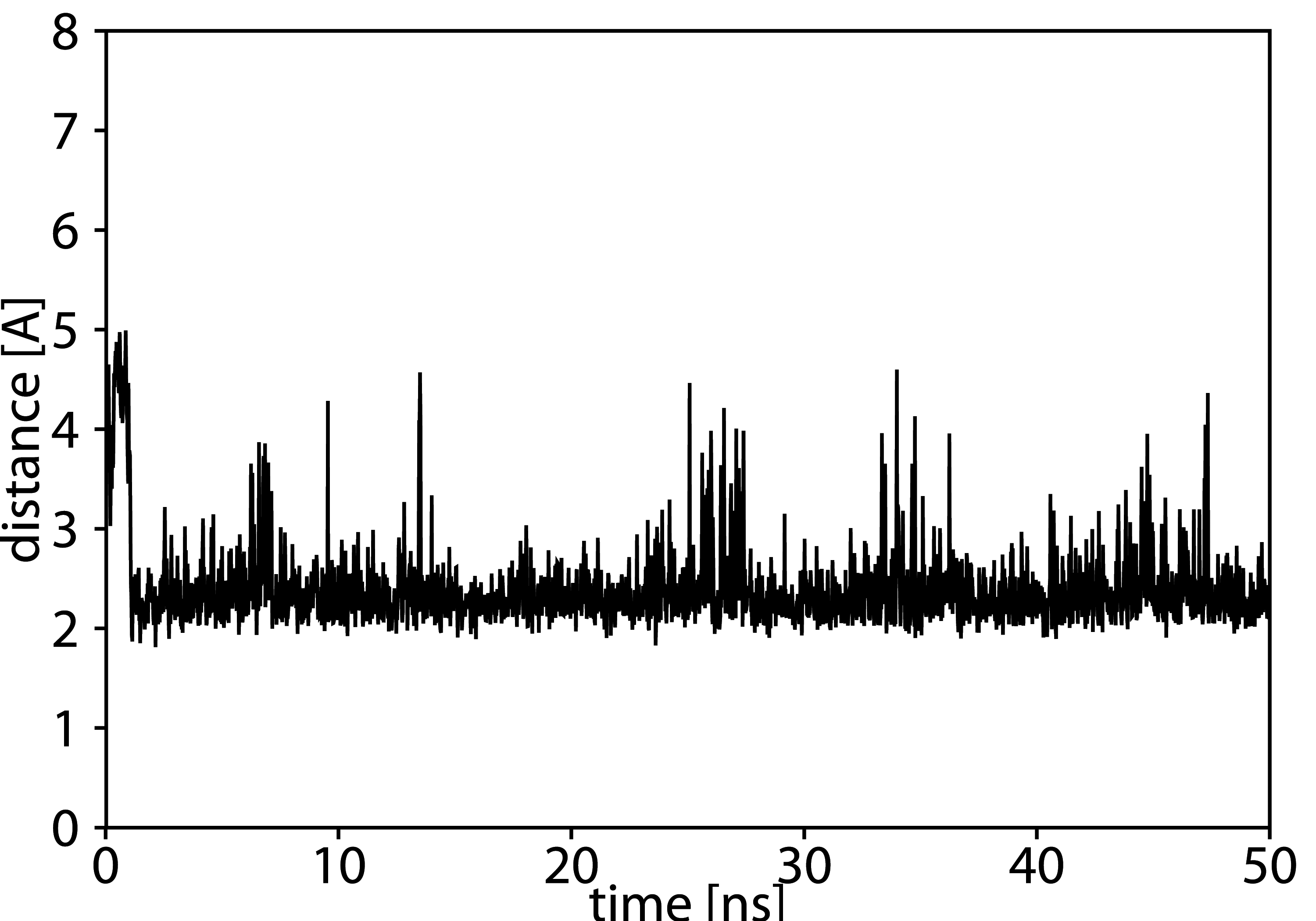

Supplement: S7 Fig — Time evolution of the distance between indicated atoms of the replica of the S2 system. (TIF) [file pone.0189588.s008.tif]

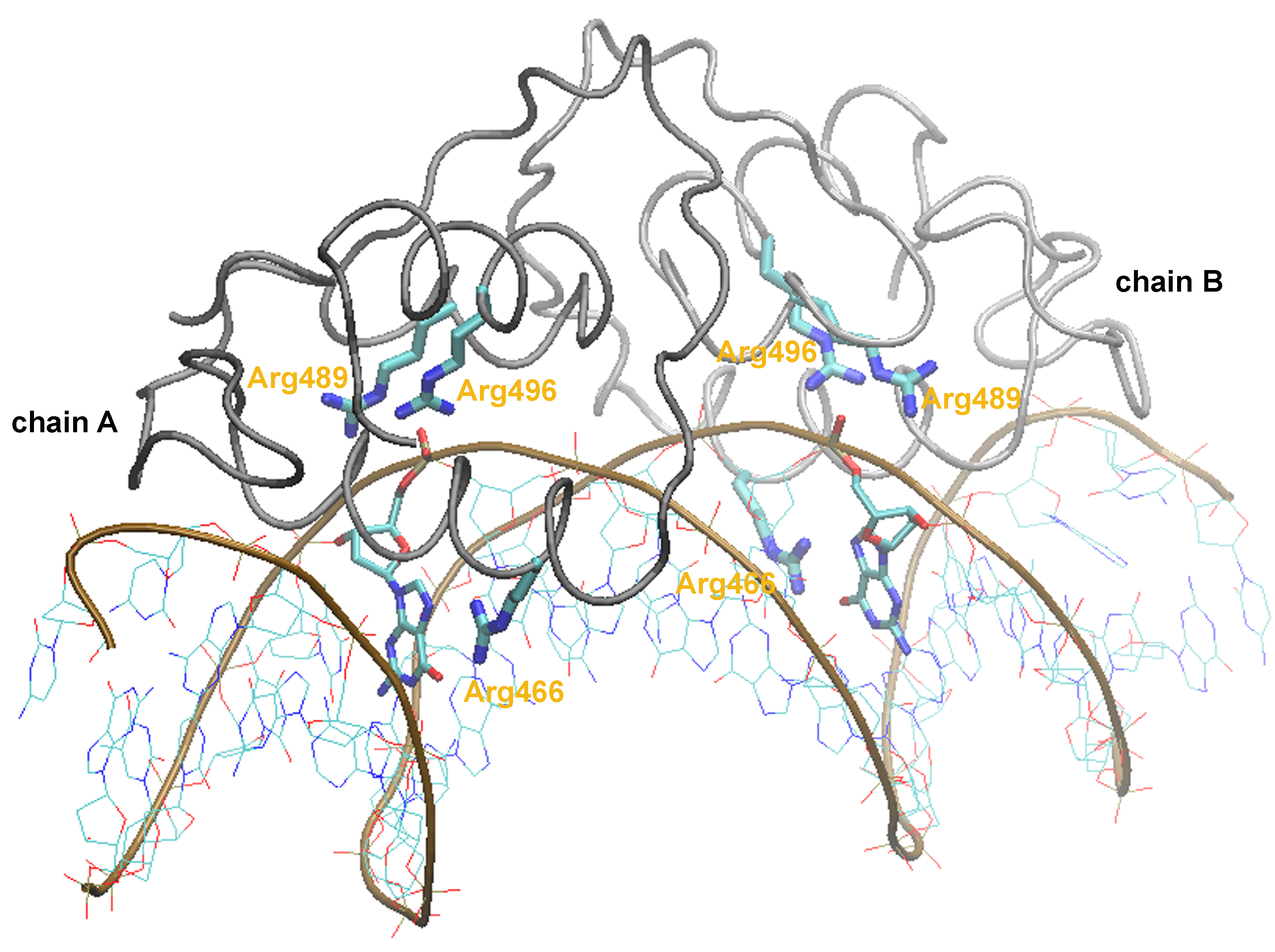

Supplement: S8 Fig — Representative structure of the S1 system showing the major polar interactions among GR DBD and DNA atoms. (TIF) [file pone.0189588.s009.tif]

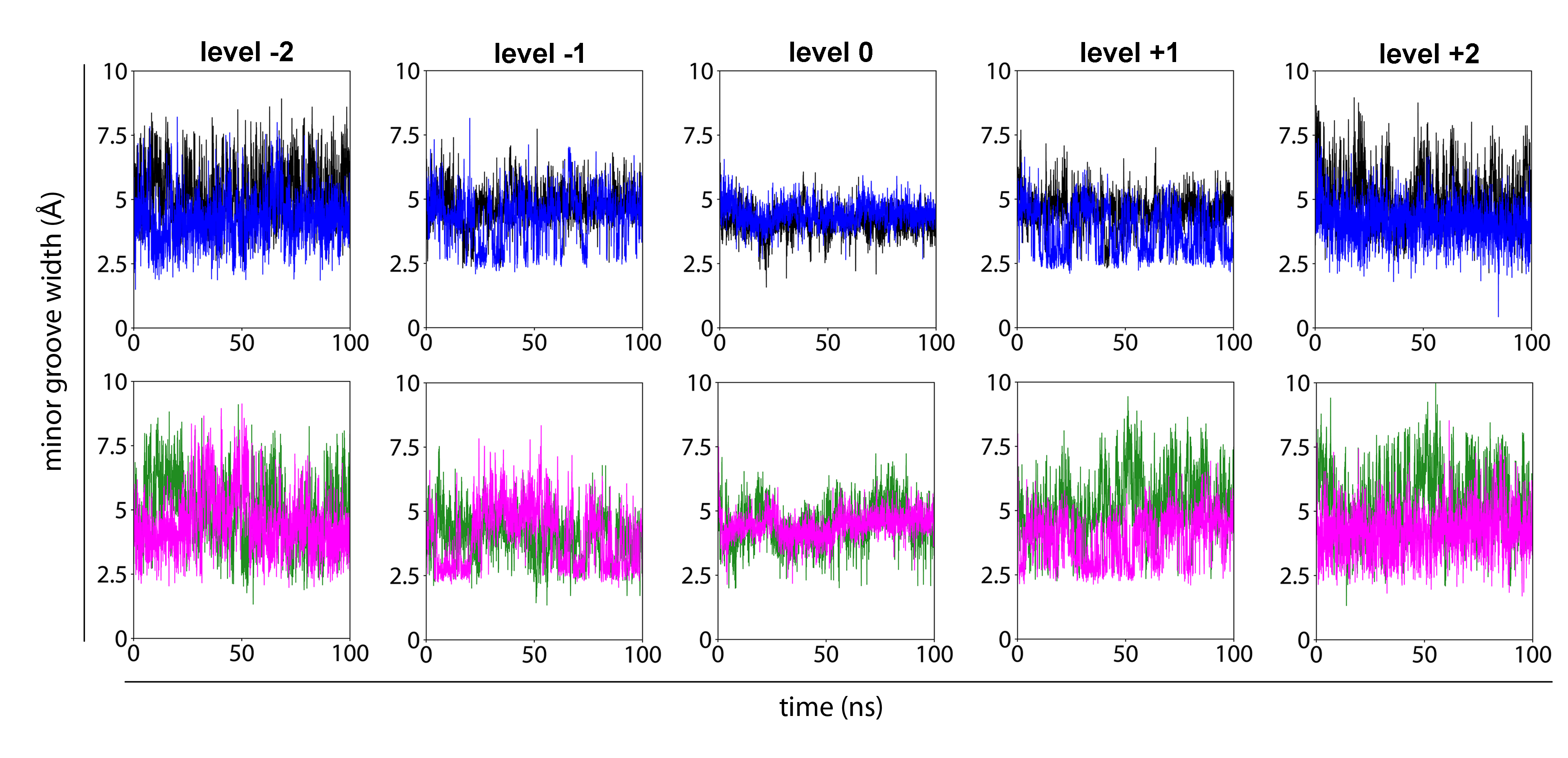

Supplement: S9 Fig — Time evolution of the minor groove width in level -2 to +2 for S1 (black), S3 (blue), S2 (green) and S4 (magenta) systems. (TIF) [file pone.0189588.s010.tif]
